# The Role of Lung Function Testing in Newborn Infants With Congenital Thoracic Arterial Anomalies

**DOI:** 10.3389/fped.2021.682551

**Published:** 2021-06-15

**Authors:** Claudia Columbo, Francesca Landolfo, Domenico Umberto De Rose, Anna Claudia Massolo, Aurelio Secinaro, Teresa Pia Santangelo, Marilena Trozzi, Cosimo Marco Campanale, Alessandra Toscano, Irma Capolupo, Pietro Bagolan, Andrea Dotta

**Affiliations:** ^1^Neonatal Intensive Care Unit, Medical and Surgical Department of Fetus, Newborn and Infant–“Bambino Gesù” Children's Hospital IRCCS, Rome, Italy; ^2^Advanced Cardiovascular Imaging Unit, Department of Imaging–“Bambino Gesù” Children's Hospital IRCCS, Rome, Italy; ^3^Airway Surgery Unit, “Bambino Gesù” Children's Hospital IRCCS, Rome, Italy; ^4^Perinatal Cardiology Unit, Medical and Surgical Department of Fetus, Newborn and Infant–“Bambino Gesù” Children's Hospital IRCCS, Rome, Italy; ^5^Newborn Surgery Unit, Medical and Surgical Department of Fetus, Newborn and Infant–“Bambino Gesù” Children's Hospital IRCCS, Rome, Italy

**Keywords:** pulmonary function tests, infants, vascular rings, aortic arch, thoracic arteries, congenital tracheal stenosis, vascular anomalies, neonate

## Abstract

**Introduction:** Congenital thoracic arterial anomalies (CTAAs), such as complete or incomplete vascular rings, pulmonary artery sling, and innominate artery compression syndrome, may cause severe tracheomalacia and upper airway obstruction. An obstructive ventilatory pattern at lung function testing (LFT) has been suggested in the presence of CTAA. The severity of obstruction may be evaluated by LFT. Little is known about the use of LFT in newborn infants with CTAA. The aim of our study is to evaluate the role of LFT in CTAA diagnosis.

**Methods:** This is a retrospective study, conducted between February 2016 and July 2020. All CTAA cases for whom LFT was performed preoperatively were considered for inclusion. Tidal volume (Vt), respiratory rate, and the ratio of time to reach the peak tidal expiratory flow over total expiratory time (tPTEF/tE) were assessed and compared to existing normative data. Demographics and CTAA characteristics were also collected.

**Results:** Thirty cases were included. All infants with CTAA showed a significantly reduced Vt and tPTEF/tE, compared to existing normative data suggesting an obstructive pattern.

No significant differences were found for LFT between cases with a tracheal obstruction <50% compared to those with tracheal obstruction ≥50%, or between cases with and without symptoms. Sixteen infants (53.3%) had respiratory symptoms related to CTAA. Of these, only two cases had also dysphagia.

**Conclusion:** LFT values were significantly reduced in cases with CTAA before surgery. LFT represents a potential feasible and non-invasive useful tool to guide diagnosis in the suspect of CTAA.

## Introduction

Congenital thoracic arterial anomalies (CTAAs) include anomalies of the aortic arch and the branches or the pulmonary arteries. CTAA may encircle the trachea and/or the esophagus resulting in an obstruction and determining respiratory symptoms such as respiratory distress, recurrent respiratory tract infections, stridor, and/or dysphagia (1). Depending on the severity of obstruction, this may determine a mild to severe respiratory distress during infancy and may require surgical treatment (1, 2). Anomalies of the aortic arch and branches are known as vascular rings (VRs).

VRs are considered as “complete” (CVR) if they have vascular structures completely encircling the trachea and the esophagus. These include the following:

1. Neuhauser's anomaly characterized by a right aortic arch (RAA) with left posterior ductus arteriosus (LPDA) arising from Kommerell diverticulum and ALSA (aberrant left subclavian artery) (RAA-LPDA-ALSA);
2. RAA with LPDA and mirror image (MI) (RAA-LPDA-MI);
3. RAA with LPDA and aberrant left innominate artery (ALIA) (RAA/LPDA/ALIA); and
4. Double aortic arch characterized by two aortic arches.

VRs are considered “incomplete” (IVR) if they only compress a portion of the esophagus or trachea without encircling it as a complete ring. These include:

1. An RAA with right ductus arteriosus (RDA) and MI (RAA/RDA/MI);
2. An RAA with left anterior ductus arteriosus (LADA) and MI (RAA/LADA/MI); and
3. A left aortic arch (LAA) with aberrant right subclavian artery (ARSA) (LAA/ARSA).

Considering similar tracheoesophageal compression, symptoms, and surgical correction, among IVRs, the other two defects involving pulmonary arteries and innominate artery can be included (3, 4):

1. Pulmonary artery sling, when the left pulmonary artery originates from the right pulmonary artery and encircles the distal trachea and right mainstem branches as it courses between the trachea and esophagus to the left lung, very commonly (almost 50% of cases) with congenital tracheal stenosis (3); and
2. Innominate artery compression syndrome when there is an abnormally distal and posterior take-off of the innominate artery and it compresses the trachea anteriorly as it courses from the left of the mediastinum to the right arm (3).

Tracheobronchomalacia (TBM) is frequently associated with these cases and explains symptoms persistence despite surgical procedures (5). The incidence of CTAAs is approximately 3%, occurring equally in both sexes, with no geographical or racial differences, although CTAAs often remain undiagnosed (6). CTAAs are usually diagnosed with cross-sectional imaging techniques [such as computed tomography (CT), magnetic resonance imaging (MRI)] and with laryngotracheobronchoscopy (LTBS) (1). However, these examinations are not easy to schedule, postponing diagnosis and treatment.

Lung function testing (LFT) is a key determinant screening tool in assessing respiratory symptoms in newborn infants (7–9). Recently, an obstructive ventilatory pattern has been suggested in the presence of CTAA (10). Therefore, LFT may represent a potential non-invasive useful screening tool for an early diagnosis in cases with suspected CTAAs. However, lack of pulmonary function laboratories, the relative complexity in performing these tests, and the need for a targeted training have limited its use.

The aims of this study are to evaluate the feasibility of LFT in an intensive care unit setting and to assess the role of LFT as a potential tool for the diagnosis of CTAA cases with and without respiratory symptoms.

## Methods

### Study Setting

We retrospectively collected data of all subjects with CTAA, prenatally or postnatally diagnosed, and then referred to “Bambino Gesù” Children's Hospital (Rome, Italy) from February 2016 to July 2020, who underwent LFT. All cases were managed by a multidisciplinary team as previously described (11). Respiratory symptoms (including inspiratory stridor, wheezing, apnea episodes, and respiratory distress) or feeding difficulties and dysphagia were recorded. Cardiac and extracardiac abnormalities and genetic testing results were also collected. Cases were either managed conservatively and monitored at follow-up or surgically treated depending on the severity of the disease (12).

### Echocardiographic Studies

All patients underwent routine echocardiography as a standard of care to evaluate suspected cases. Fetal and postnatal echocardiography was carried out using a Philips IE 33 ultrasound machine with a 5–2-MHz convex transducer and 8–3- and/or 12–4-MHz sector transducers, or a Philips Epiq 7G ultrasound machine with a 9–2-MHz convex transducer and 8–3- and/or 12–4-MHz sector transducers (Philips, Andover, MA, USA) as previously described (13).

### Computed Tomography

A CT scan was performed to confirm the diagnosis and to measure the percentage of tracheal obstruction. CT was performed in all subjects with contrast enhancement during inspiration and expiration phases without general anesthesia to assess vascular malformations and their relationship with close structures; image analysis was performed on an offline workstation (Multimodality Workplace, Siemens Healthcare) as previously reported (14). A grading system of tracheal stenosis was used to stratify tracheal stenosis in four grades (grade I: stenosis up to 50%; grade II: stenosis between 51 and 70%; grade III: stenosis >70%; grade IV: no lumen visualized at the narrowest point) (15). Dynamic pulmonary CT was adopted to detect excessive collapsibility of trachea and/or bronchi: the presence of TBM was defined on video recordings as ≥50% reduction in the airway lumen during expiration, as previously described (16).

### Laryngotracheobronchoscopy

Most subjects underwent preoperative LTBS as part of a standardized preoperative diagnostic protocol to assess the presence of vascular pulse on the trachea and the percentage of tracheal obstruction. The diagnostic procedure was performed using a rigid scope 4 mm 0° under general anesthesia in tubeless spontaneous ventilation (17). The cardiothoracic surgeon often asked for endoscopic visual support at the end of surgery on the vessels before the closure of surgical access to confirm the resolution of compression on the airways. Intraoperative endoscopy is always performed with a flexible instrument often through the externalized endotracheal tube or without a tube when the patient is in extracorporeal circulation.

### Lung Function Testing

An ultrasonic flowmeter (Spiroson, ndd Medical Technologies, Zurich, Switzerland) connected to an Exhalyzer D (Eco Medics, Sensormedics, Bern, Switzerland) was used to perform tests. Calculations were automatically analyzed by Spiroware WBreath software version 2.0 (ndd Medical Technologies) (8, 9). Data were collected by experienced operators to analyze LFT (CC and FL) and identify the severity of pulmonary impairment. All measurements were performed in neutral supine position during spontaneous sleep, as previously described (8, 9, 18). Airway flow volume loops were recorded and analyzed during inspiration and expiration. A minimum of 10 regular breaths were recorded. Measurements collected were tidal volume (Vt), respiratory rate, the ratio of time to reach peak tidal expiratory flow over total expiratory time (tPTEF/tE), and tidal expiratory flow related to tidal inspiratory flow, both at 50% volume). LFT measurements were identified as previously described (19).

### Statistical Analysis

We compared lung function values of infants with CTAA to existing normative values provided by Fuchs et al. from acceptable tidal breathing measurements in 285 infants at 5 weeks of age (19). Data of cases referred to our center for suspected VR but without evidence of it could not be used as a healthy comparison group for the presence of respiratory symptoms.

We also compared lung function values between infants with CTAA with tracheal obstruction <50% and ones with tracheal obstruction ≥50% and between infants with or without symptoms.

Data are presented as numbers and percentages for categorical variables. Continuous variables are expressed as mean ± SD or median (range), depending on the distribution of the variable (evaluated with D'Agostino–Pearson test). Groups were compared with Fisher test, *t*-test, or Mann–Whitney *U*-test as appropriate. *p* < 0.05 was considered significant. Data were analyzed with MedCalc Software package for Windows, release 12.7 (MedCalc Software, Belgium).

### Ethical Statement

All procedures performed in this study were in accordance with the ethical standards of the institutional and national research committee and with the 1964 Helsinki Declaration and its later amendments or comparable ethical standards. The study was provided by our Scientific Directorate, and as a retrospective analysis with no patient-identifiable information, it was approved without need for written consent. Personal data were restricted to essential information and were treated in order to guarantee the respect of privacy of the involved patients, as specifically stated by Italian Law D. Lgs. n.196 of 2003 about personal data protection.

## Results

### Demographics and Characteristics

Thirty cases with CTAA who underwent LFT preoperatively during the study period were included. Infants' characteristics and type of CTAA are summarized in Table 1. In 23 pregnancies, karyotyping was declined or not performed, but none of these had features suggestive of a chromosomal abnormality in the prenatal and/or postnatal records. Of the 30 cases, two had a confirmed genetic anomaly: one had Down syndrome with patent ductus arteriosus, portocaval shunt (Abernethy type 1), and lung emphysema, and one had DiGeorge syndrome due to 22q11.2 deletion with ventricular septal defect, hypocalcemia, hypothyroidism, left choanal stenosis, clubfeet, and palatoschisis. Among the other observed anomalies, one had a ring-sling complex type 2A, a ventricular septal defect, and a bridging bronchus; two had esophageal atresia; and one had tetralogy of Fallot. Only 18 subjects (60%) had a prenatal diagnosis of CTAA: other infants were referred because of symptoms or other anomalies (Table 1). There were no significant differences for age at surgical repair between infants with prenatal or postnatal diagnosis (*p* = 0.6). Demographics were similar between cases with CVR or IVR; no significant differences were found.

**Table 1:** Demographics.

	**Cases (*n*= 30)**
Gestational age, weeks, mean (SD)	38.0 (2.0)
Weight at birth, g, median (range)	3,090 (1,450–3,900)
Males, *n* (%)	22 (73.3)
Prenatally diagnosed, *n* (%)	18 (60.0)
Symptoms related to CTAA, *n* (%)	16 (53.3)
Age at the time of the LFT, days, median (range)	114 (3–421)
Weight at the time of the LFT, grams, median (range)	5,900 (2,300–10,800)
Surgical repair, *n* (%)	14 (46.7)
Age at surgical repair, days, median (range)	100 (64–120)
Complete vascular ring, *n* (%)	22 (73.3)
Percentage of tracheal obstruction 0–50%, *n* (%); 50–70%, *n* (%); >70%, *n* (%); no lumen, *n* (%)	19 (63.3), 5 (16.7), 5 (16.7), 1 (3.3)
Type of CTAA, *n* (%)	1 (3.3) PAS, 6 (20) DAA, 13 (43.3) RAA-LPDA-ALSA, 1 (3.3) LAA-ARSA, 6 (20) IACS, 3 (10) RAA-LPDA-MI
Symptoms in 18 infants with prenatal diagnosis, *n* (%)	3 (16.7) prenatal diagnosis and stridor 1 (5.6) prenatal diagnosis and respiratory distress 1 (5.6) prenatal diagnosis and wheezing 13 (72.2) prenatal diagnosis only
Symptoms in 12 infants with postnatal diagnosis, *n* (%)	3 (25.0) stridor 1 (8.3) stridor and dysphagia 1 (8.3) stridor and respiratory distress 3 (25.0) respiratory distress 2 (16.7) wheezing 1 (8.3) dysphagia (postnatal diagnosis of esophageal atresia) 1 (8.3) postnatal diagnosis of tetralogy of Fallot

*SD, standard deviation; g, grams; n, number; CVR, complete vascular ring; IVR, incomplete vascular ring, CTAA, congenital thoracic arterial anomalies; PAS, pulmonary artery sling; DAA, double aortic arch; RAA, right aortic arch; LAA, left aortic arch; LPDA, left posterior ductus arteriosus; ALSA, aberrant left subclavian artery; ARSA, aberrant right subclavian artery; IACS, innominate artery; MI, mirror image*.

### Lung Function Testing

**Figure 1:**
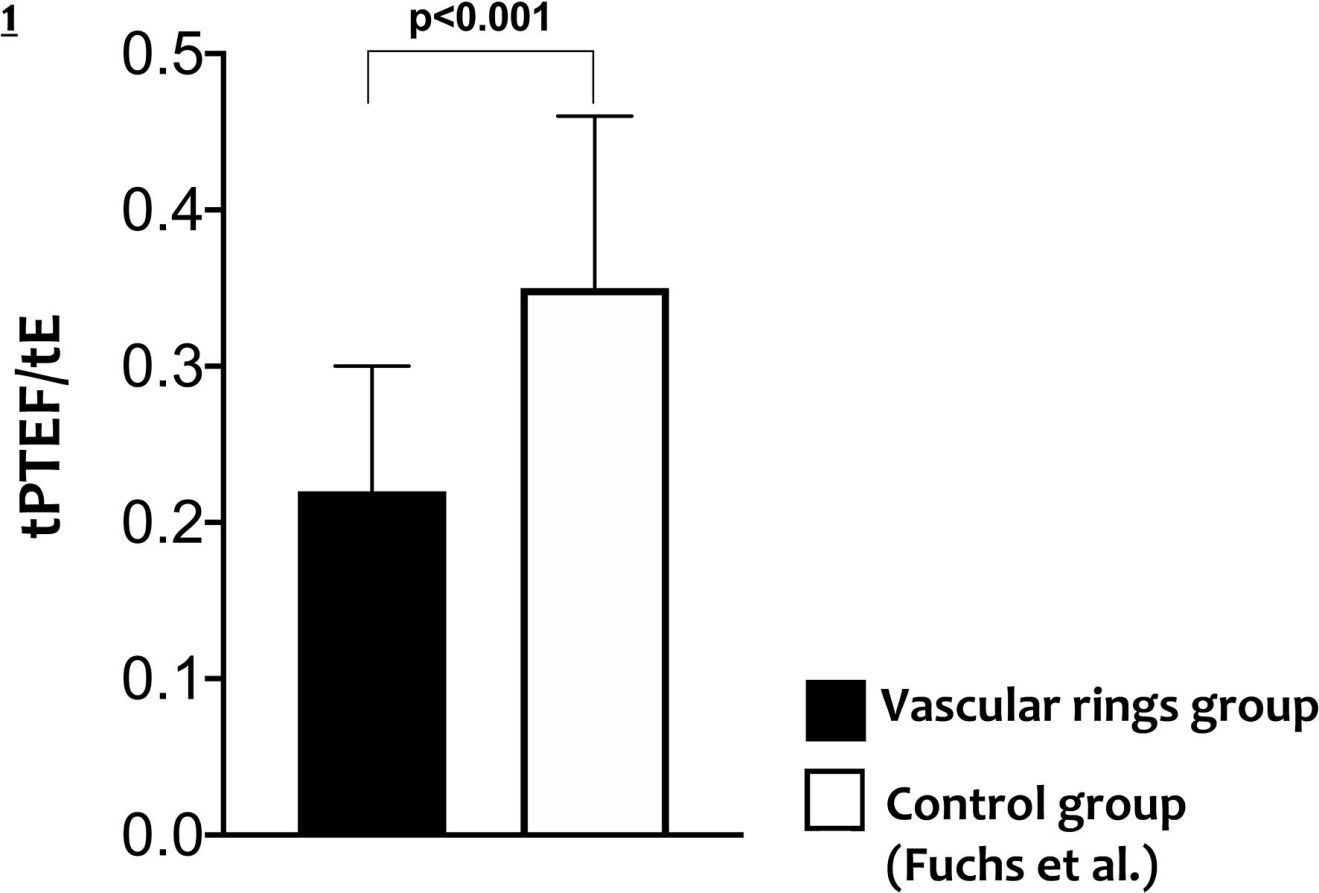
Differences in tPTEF/tE in cases with vascular rings and in controls.

All infants with CTAA had significantly lower Vt and ratio of time to reach the peak tidal expiratory flow over total expiratory time (tpTEF/tE) when compared to existing normative lung function values (Table 2, Figure 1).

**Table 2:** LFT measurements in infants with CTAA compared to normative lung function values.

	**Infants with CTAA (*n* = 30)**	**Normative lung function values by Fuchs et al. (20) (*n* = 285)**	***p*-value**
Vt, in ml/kg, mean (SD)	5.64 (2.87)	7.48 (1.29)	** <0.001**
Respiratory rate, breaths/minute, mean (SD)	45.5 (13.3)	45.2 (10.5)	0.9
tPTEF/tE, mean (SD)	0.22 (0.08)	0.35 (0.11)	** <0.001**

*Vt, tidal volume; tPTEF/Te, ratio of time to reach the peak tidal expiratory flow over total expiratory time; CTAA, congenital thoracic arterial anomalies; SD, standard deviation. Bold values are related to statistically significant values*.

**Figure 2:**
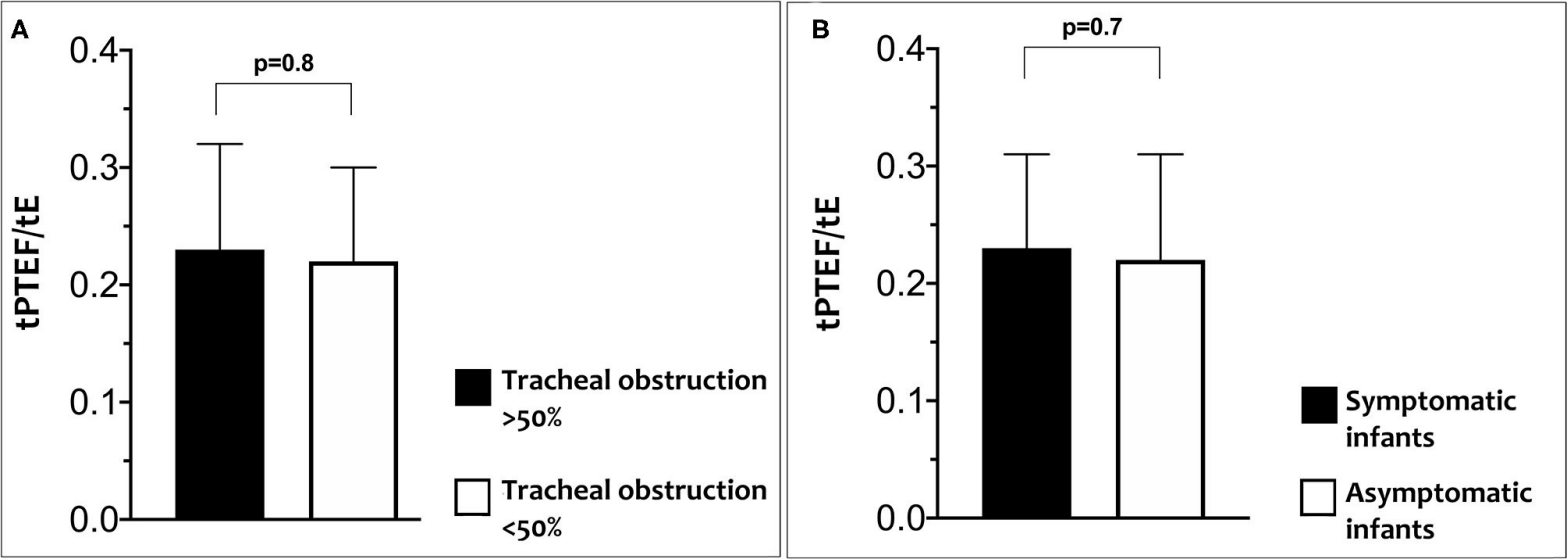
Differences in tPTEF/tE according to percentage of tracheal obstruction **(A)** and in tPTEF/tE according to related symptoms **(B)**.

No significant differences were found for LFT between cases with a tracheal obstruction <50% compared to those with tracheal obstruction ≥50% or between cases with and without symptoms (Figures 2A,B).

**Figure 3:**
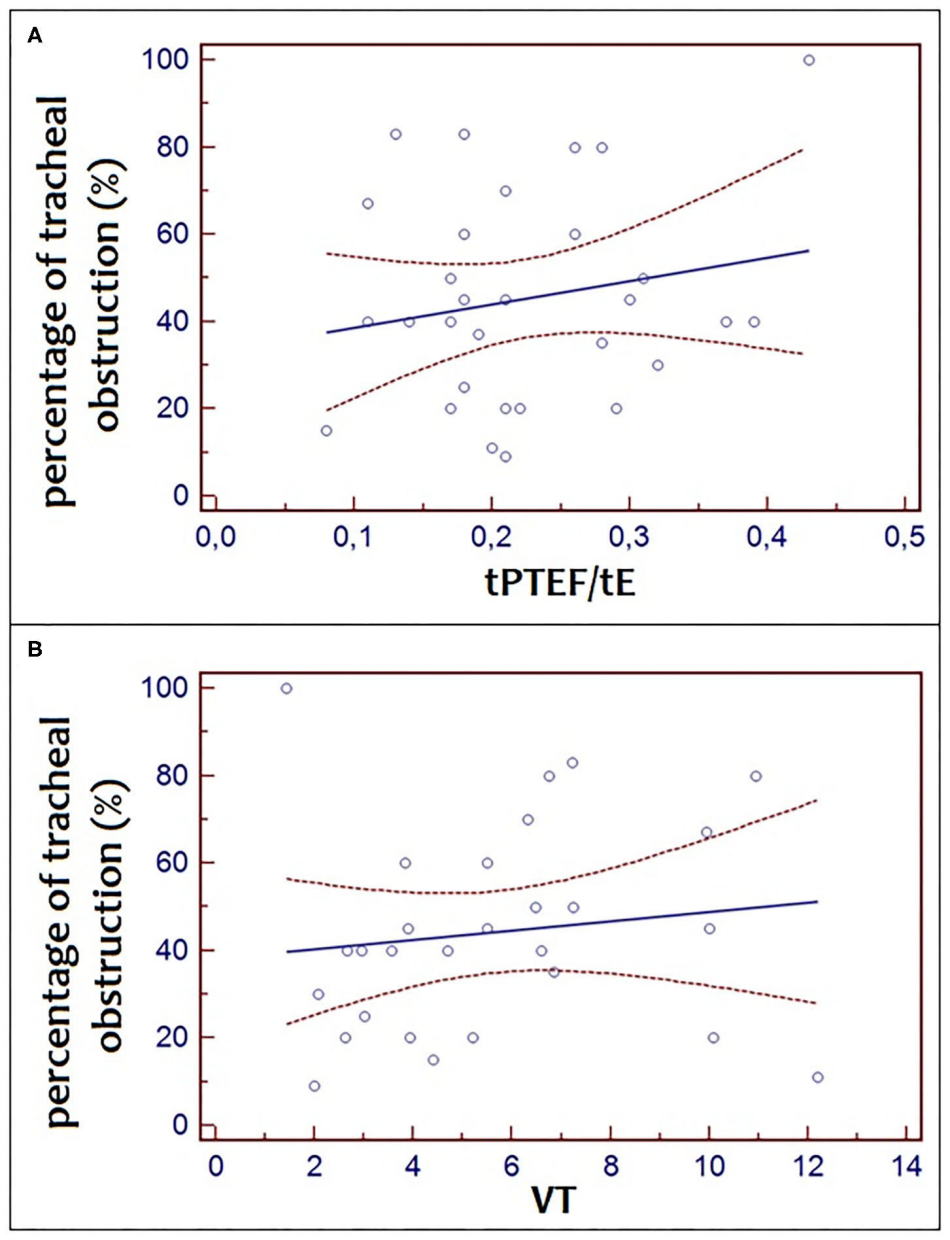
Correlation between the percentage of tracheal obstruction and tPTEF/tE (*r*^2^ = 0.04, *p* = 0.32) and between the percentage of tracheal obstruction and VT (*r*^2^ = 0.06, *p* = 4 0.22).

There was no significant correlation between the percentage of tracheal obstruction and tPTEF/tE (*r*^2^ = 0.04, *p* = 0.32) and between the percentage of tracheal obstruction and VT (*r*^2^ = 0.06, *p* = 0.22) (Figure 3).

### Symptoms, Tracheal Obstruction, and Tracheobronchomalacia

Sixteen infants (53.3%) had symptoms related to CTAA, of whom only two cases (12.5%) had dysphagia, but all had respiratory symptoms. Main respiratory symptoms were stridor, respiratory distress syndrome, and wheezing in eight (50%), five (31.3%), and three (18.8%) cases, respectively.

Cases who had symptoms significantly more often underwent surgical repair compared to those who did not [11 (68.8%) vs. 3 (21.4%) respectively, *p* = 0.01].

Percentage of obstruction evaluated with a CT scan was classified in four grades of increasing degree of severity as discussed above and are summarized in Table 1. No significant differences were found for LFT between different grades of tracheal obstruction or between infants with and without symptoms.

Twenty-four subjects underwent dynamic expiratory CT: of these, nine (37.5%) had TBM. LTBS was performed in 28 subjects. No significant differences in the grade of tracheal obstruction measured by CT or LTBS were found.

## Discussion

In this study, LFT was performed in a cohort of newborn infants with CTAAs. We found a lower Vt and ratio of time to reach peak tidal expiratory flow over total expiratory time (tPTEF/tE) in all infants with CTAA, suggesting an obstructive pattern compared to existing normative data.

Accordingly, previous case report studies described an increased intrathoracic obstruction in infants and children with VRs, suggesting increased expiratory airway resistance and decreased maximal expiratory flows (10, 21–23). A CVR that determines an intrathoracic tracheal obstruction showed a plateau of both inspiratory and expiratory flows, whereas an IVR was associated solely to a plateau of the expiratory flow (24).

**Figure 4:**
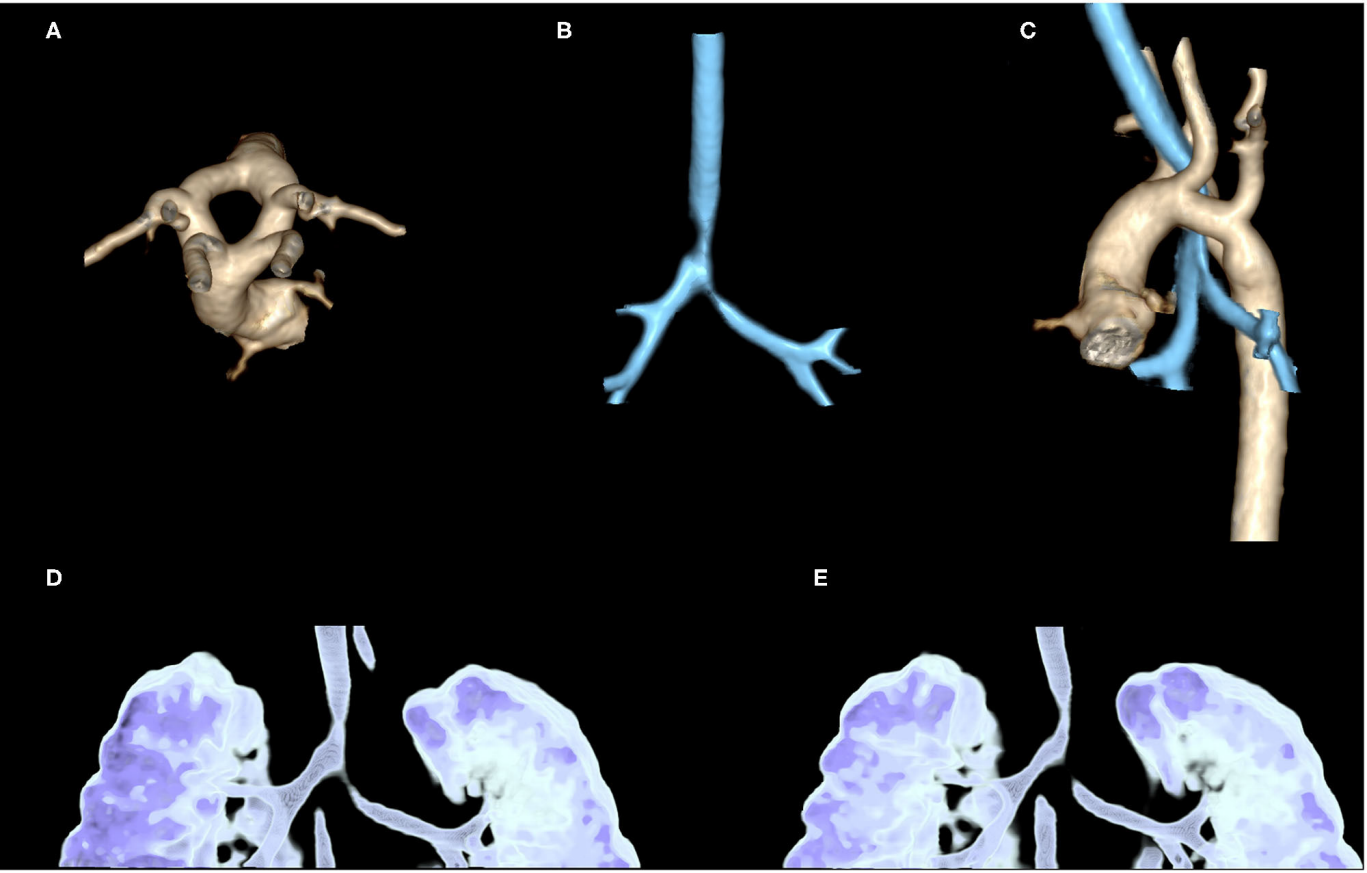
Contrast-enhanced CT in a 2-month-old girl, referred to our hospital after prenatal diagnosis of vascular ring: three-dimensional (3D) volume rendering of thoracic aorta confirms the presence of a double aortic arc with two arches (left and right) equally sized **(A)**. 3D volume rendering of airways shows stenosis of distal trachea until carina, near the vascular ring **(B)**. An evaluation of these stenoses compared to thoracic aorta shows that they are, respectively, at the level of the vascular ring and left arterial duct **(C)**. 3D volume rendering of the dynamic expiratory phase displays associated signs of tracheobronchomalacia of distal trachea and left-sided bronchus with significant variation of caliber between the inspiratory **(D)** and expiratory phase **(E)**.

CTAA may determine different clinical features and sometimes be confused with other diseases such as wheezing, gastroesophageal reflux, idiopathic stridor, and dysphagia. In some cases, the diagnosis may be delayed for several years, particularly in those with less severe symptoms. Therefore, in cases with persistent upper airway obstruction, VRs should always be excluded especially when a prenatal diagnosis is not available (13, 23). CT scan allows diagnosis and a more accurate definition of the airways and foregut malformations, even with a three-dimensional approach (Figure 4), particularly for its higher spatial resolution, compared to MRI. However, CT exposes the patient to ionizing radiation, which is a relevant concern in pediatric subjects and is not always available or easy to schedule, resulting in delayed diagnosis (14, 25).

Supporting our findings, a tPTEF/tE reduction has been suggested as a more direct indicator of airway obstruction in a cohort of preterm infants with and without bronchopulmonary dysplasia, suggesting that LFTs may help to identify those infants at greater risk of severe respiratory morbidity (26). Similarly, LFTs may represent an adjunct as a useful bedside tool that can help clinicians to screen infants with a suspected VR, who need a further invasive examination.

Based on our findings, LFTs are not associated with disease severity, although we speculate that LFTs may be combined with other bedside tools such as myocardial strain. Myocardial strain, evaluated by echocardiography, has been associated with several diseases in critically ill newborn infants (27–29). Previous studies conducted by our team showed a reduction in myocardial strain in infants with VR, particularly in those with a tracheal obstruction >50%, suggesting a secondary cardiac dysfunction related to severe tracheomalacia (12). Moreover, the severity of myocardial impairment correlates with the severity of the airway obstruction suggesting that the combination with LFT may support clinicians during clinical decision-making and follow-up, particularly in those cases with mild symptoms.

In our clinical setting, LFT evaluation is routinely used as a first screening in infants with upper airway obstruction. Postoperative LFTs assessment was not performed in the current cohort due to an absence of postoperative LFTs; however, a prospective investigation of the changes between preoperative and postoperative LFT is in progress.

The main limitations of this study were its retrospective single-center design and the small sample size. This may also explain the lack of association between LFT and disease severity. CTAAs remain rarely identified in neonatal age; thus, this cohort may be considered an appropriate sample. Infants' ages differ between the control group and the VR group; thus, all measures were corrected by the patients' weight in order to standardize all measurements.

Several studies demonstrated the feasibility of LFT in children and infants with respiratory symptoms, although LFT equipment and procedures may be considered complex, and recommendations for professionals conducting pulmonary function tests exist (30–34).

Our findings suggest that LFTs are feasible in critically ill newborn infants with suspected CTAA. An obstructive pattern characterizes infants with VRs, both in cases with and those without symptoms. LFTs should be considered in case of suspected CTAA and may represent a useful tool to guide clinicians for early diagnosis and treatment improving outcomes. Future prospective studies, on larger sample size, also investigating lung function after surgery and including a healthy control group, are needed to confirm these findings.

## Data Availability Statement

The original contributions presented in the study are included in the article/supplementary material, further inquiries can be directed to the corresponding author/s.

## Ethics Statement

Ethical review and approval was not required for the study on human participants in accordance with the local legislation and institutional requirements. Written informed consent from the participants' legal guardian/next of kin was not required to participate in this study in accordance with the national legislation and the institutional requirements.

## Author Contributions

CC and FL conceptualized and designed the study, designed the data collection instruments, performed lung function tests, enrolled subjects, collected data, contributed to the interpretation of the results, reviewed, and revised the manuscript. DD performed literature search, designed the data collection instruments, collected data, analyzed data, and drafted the initial manuscript. AM performed literature search, reviewed, and revised the manuscript. AS and TS performed computed tomography, collected data, reviewed, and revised the manuscript. MT performed laryngotracheobronchoscopy, collected data, reviewed, and revised the manuscript. CMC and AT performed echocardiography, collected data, reviewed, and revised the manuscript. IC, PB, and AD coordinated and supervised data collection, and critically reviewed the manuscript for important intellectual content. All authors approved the final manuscript as submitted and agree to be accountable for all aspects of the work.

## Conflict of Interest

The authors declare that the research was conducted in the absence of any commercial or financial relationships that could be construed as a potential conflict of interest.

## Abbreviations

ALIA: aberrant left innominate artery
ALSA: aberrant left subclavian artery
ARSA: aberrant right subclavian artery
CT: computed tomography
CTAA: congenital thoracic arterial anomalies
CVR: complete vascular ring
IVR: incomplete vascular ring
LAA: left aortic arch
LADA: left anterior ductus arteriosus
LFT: lung function testing
LPDA: left posterior ductus arteriosus
LTBS: laryngotracheobronchoscopy
MI: mirror image
MRI: magnetic resonance imaging
RAA: right aortic arch
RDA: right ductus arteriosus
RR: respiratory rate
TBM: tracheobronchomalacia
tPTEF/tE: ratio of time to reach the peak tidal expiratory flow over total expiratory time
VR: vascular rings
Vt: tidal volume.
